# Obesity classification: a comparative study of machine learning models excluding weight and height data

**DOI:** 10.1590/1806-9282.20241282

**Published:** 2025-03-17

**Authors:** Ahmed Cihad Genc, Erkut Arıcan

**Affiliations:** 1 Bahcesehir University, Graduate School, Department of Artificial Intelligence – İstanbul, Türkiye.; 2 Sakarya University, Faculty of Medicine, Department of Internal Medicine – Sakarya, Türkiye.; 3 Bahcesehir University, Department of Computer Engineering, İstanbul, Türkiye.

**Keywords:** Artificial intelligence, Classification, Machine learning, Obesity

## Abstract

**OBJECTIVE:**

Obesity is a global health problem. The aim is to analyze the effectiveness of machine learning models in predicting obesity classes and to determine which model performs best in obesity classification.

**METHODS:**

We used a dataset with 2,111 individuals categorized into seven groups based on their body mass index, ranging from average weight to class III obesity. Our classification models were trained and tested using demographic information like age, gender, and eating habits without including height and weight variables.

**RESULTS:**

The study demonstrated that when trained on demographic information, machine learning can classify body mass index. The random forest model provided the highest performance scores among all the classification models tested in this research.

**CONCLUSION:**

Machine learning methods have the potential to be used more extensively in the classification of obesity and in more effective efforts to combat obesity.

## INTRODUCTION

Across the world, the two approaches that are mostly used to determine obesity include waist circumference and body mass index (BMI) screenings. BMI is an index directly calculated using the variables of height and weight^1^. BMI is ubiquitous in clinical practice due to its ease of use. It correlates with increased body weight as well as levels of body fat. BMI is used as an indirect and easily obtained marker to get body fat and usually gives an even better estimation of fat than weighing^2^. The prevalence of obesity has increased rapidly in the last decade, making it a public health problem that also increases comorbidities such as diabetes, hypertension, and stroke^3^. BMI effectively categorizes individuals into seven distinct levels, beginning with insufficient weight for BMI below 18.5, followed by normal weight ranging from 18.5 to 24.9, overweight level I between 25 and 29.9, overweight level II from 30 to 34.9, obesity type I from 35 to 39.9, obesity type II ranging from 40 to 44.9, and continuing with obesity type III for BMI values of 45 and above^4^. Comorbidities and severity vary depending on the obesity level^5^.

Artificial intelligence has gained global recognition and includes machine learning (ML), which utilizes sophisticated neural networks. ML can be categorized into two primary types, with unsupervised learning functioning without labeled data, while supervised learning relies on labeled data for guidance^6^.

Among the ML models that perform classification, one is based on logistic regression^7^. Logistic regression is a statistical method employed to predict the likelihood of a specific outcome by analyzing one or more predictor variables^8^. Classification and regression trees are collective under the tree-based model. They stand out for their readability and can be used on numerical and categorical data^9^. Random forests, which belong to the ensemble learning techniques, are quite useful in supervised learning due to their high accuracy and efficiency in handling large datasets with many features^10^. Support vector machines (SVMs) are highly efficient for classification and regression, particularly in handling numerous features and complex nonlinear relationships between features and targets^11^. Further mathematical development describes SVMs and, finally, the ways in which, guided by inside support vectors, they maximize the entire width margin that separates the classes^12^. K-Nearest Neighbors (KNN) assigns labels to unlabeled data points based on their nearest neighbors in a labeled dataset and, despite not requiring knowledge of joint distributions, can achieve an error probability at least as low as the Bayes error rate, with the potential for twice the Bayes error rate in multi-category situations. This indicates its effectiveness even with small sample sizes^13^. KNN is also one of the most conventional nonparametric regression techniques because of its ease of use and high accuracy^14^. The KNN algorithm is a simple technique that depends on k nearest data points of a given dataset to predict new observations^15^. Naive Bayes is a straightforward but effective probabilistic classifier that uses Bayes’ theorem to classify events and predict them even when the assumption of feature independence is almost false^16,17^. Some of the most essential AI techniques are neural networks, which mimic the individual's brain and help learn and adapt to various tasks in dealing with data^18,19^.

### Aim

This study investigates the capabilities of ML models to predict obesity and its levels without using height and weight variables, thereby improving obesity management and prevention strategies.

## METHODS

**Figure 1:**
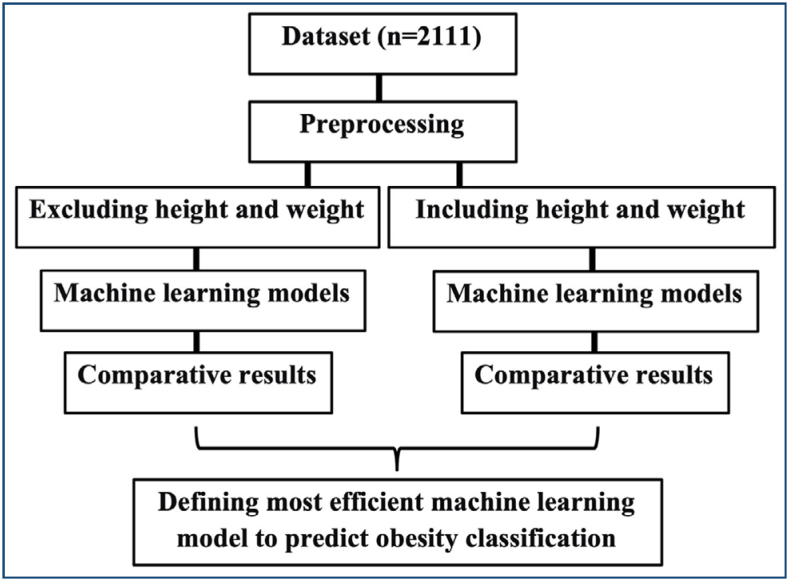
Flowchart of the study.

A total of 2,111 individuals, categorized into seven groups based on BMI, form the open-access obesity dataset from Peru, Mexico, and Colombia, which estimates obesity classes using physical and dietary indicators^20^. The dataset contains 17 variables, such as age, gender, height, weight, family history of overweight, frequency of physical activity (FAF), frequent consumption of high-caloric food (FAVC), daily consumption of water (CH_2_O), frequent consumption of vegetables (FCVC), number of meals per day (NCP), smoking, consumption of alcohol (CALG), consumption of food between meals (CAEC), caloric consumption monitoring (SCC), time using technology devices (TAU), type of transportation used (MTRANS), and obesity classes (NObeyesdad). Obesity as a target variable is classified into different categories, including underweight, average weight, overweight (Level I and Level II), and obesity (Type I, Type II, and Type III). The flowchart of the study is visualized in Figure 1.

### Data preprocessing

The dataset undergoes thorough preprocessing, including handling missing values, encoding categorical variables, normalizing and scaling numerical data, and selecting key features to improve model performance. Additionally, outlier detection and treatment are performed to ensure data accuracy, with adjustments to mitigate any undue influence on the analysis.

### Machine learning model

A 20-fold cross-validation is applied, where the dataset is split into 20 parts, and the model is trained and tested 20 times. Each subset is used once as validation data to prevent overfitting, and the final performance estimate is obtained by averaging the results from all rounds. The two most influential parameters in the model are identified using One Rule (OneR). Seven different ML models are applied to the classification tasks. Seven different ML models are applied to the classification tasks. ML classification models such as decision trees, SVM, KNN, neural networks, logistic regression, random forest, and Naive Bayes have been applied. Specific settings such as batch size, base classifiers, distance metrics, and kernel functions are adjusted to enhance model performance across different algorithms.

### Used software and statistical analysis

Various ML methods are applied to classify a seven-category variable using WEKA, version 3.9.6, and Python, version 3.8.10. Libraries such as sci-kit-learn and Pandas are used for data manipulation and analysis, and tools like Matplotlib are used for result visualization. Descriptive analyses, including visual and analytical techniques, summarize the characteristics of the study population. The numerical data are expressed as mean and standard deviations, and the categorical data are expressed as frequencies and proportions.

## RESULTS

The study population consists of 1,068 males (50.6%) and 1,043 females (49.4%), with a mean age of 24.3±6.3 years. The mean height is 1.7±0.09 m, and the mean weight is 86.6±26.2 kg. A family history of overweight is present in 81.7% of participants, and 88.4% report FAVC. The NCP is 2.69±0.78, and the mean CH_2_O is 2±0.6 L. The FAF is low, with a mean of 1.01±0.85, while the mean TAU is 0.66±0.60 h. Public transportation is the most common mode of transport, used by 74.9% of the study population. Smoking history is reported by 2.1% of individuals. Regarding BMI classifications, 12.9% of the study population falls into the insufficient weight category, 13.6% falls into the normal weight category, and the remainder are classified as overweight or obese, with Obesity Type I being the most prevalent at 16.6% (Table 1).

**Table 1:** Descriptive statistics of the study population of 2,111 people: Demographic characteristics, behavioral characteristics, and body mass index classifications.

Variable		Mean±SD/n (%) n=2,111
Age, years		24.3±6.3
Gender		
	Male	1,068 (50.6%)
	Female	1,043 (49.4%)
Height, m		1.7±0.09
Weight, kg		86.6±26.2
Family history of overweight, yes		1,726 (81.7%)
Frequent consumption of high-caloric food (FAVC), yes		1,866 (88.4%)
Frequent consumption of vegetables (FCVC), frequent		2.42±0.534
Number of meals per day (NCP), number		2.69±0.78
Consumption of food between meals (CAEC)		
	Sometimes	1,765 (83.6%)
	Frequently	242 (11.5%)
	Always	53 (2.5%)
	No	51 (2.4%)
Smoking history, yes		44 (2.1%)
Daily consumption of water (CH_2_O), L		2±0.6
Caloric consumption monitoring (SCC), yes		96 (4.5%)
Frequency of physical activity (FAF), frequency		1.01±0.85
Time using technology devices (TAU), h		0.66±0.60
Consumption of alcohol (CALG)		
	No	639 (30.3%)
	Sometimes	1,401 (66.4%)
	Frequently	70 (3.3%)
	Always	1 (<0.1%)
Type of transportation used (MTRANS)		
	Public transportation	1,580 (74.9%)
	Walk	56 (2.7%)
	Automobile	457 (21.6%)
	Motorbike	11 (0.5%)
	Bike	7 (0.3%)
Body mass index (BMI) classes		
	Insufficient weight	272 (12.9%)
	Normal weight	287 (13.6%)
	Overweight level I	290 (13.7%)
	Overweight level II	290 (13.7%)
	Obesity type I	351 (16.6%)
	Obesity type II	297 (14.1%)
	Obesity type III	324 (15.4%)

SD: standard deviation; FAVC: frequent consumption of high-caloric food; FCVC: frequent consumption of vegetables; NCP: number of meals per day; CAEC: consumption of food between meals; SCC: caloric consumption monitoring; FAF: frequency of physical activity; TAU: time using technology devices; CALG: consumption of alcohol; MTRANS: type of transportation used; BMI: body mass index.

The OneR model with all variables has an Area Under the Receiver Operating Characteristic Curve (ROC area) of 0.81, a sensitivity of 0.67, a specificity of 0.94, a precision of 0.67, and a recall of 0.67. Without height and weight variables, the ROC area is 0.67, the sensitivity is 0.44, the specificity is 0.90, the precision is 0.42, and the recall is 0.44. The logistic regression classification model with all variables achieves an ROC area of 0.98, a sensitivity of 0.84, a specificity of 0.97, a precision of 0.84, and a recall of 0.84. Without height and weight variables, the ROC area is 0.91, the sensitivity is 0.66, the specificity is 0.94, the precision is 0.65, and the recall is 0.66. The decision tree model with all variables shows an ROC area of 0.98, a sensitivity of 0.94, a specificity of 0.99, a precision of 0.94, and a recall of 0.94. Without height and weight variables, the ROC area is 0.90, the sensitivity is 0.79, the specificity is 0.96, the precision is 0.79, and the recall is 0.79. The SVM model with all variables reports an ROC area of 0.96, a sensitivity of 0.85, a specificity of 0.97, a precision of 0.85, and a recall of 0.85. Without height and weight variables, the ROC area is 0.86, the sensitivity is 0.63, the specificity is 0.94, the precision is 0.63, and the recall is 0.63. The KNN model with all variables shows an ROC area of 0.90, a sensitivity of 0.82, a specificity of 0.97, a precision of 0.82, and a recall of 0.82. Without height and weight variables, the ROC area is 0.88, the sensitivity is 0.78, the specificity is 0.96, the precision is 0.78, and the recall is 0.78. The Naive Bayes model with all variables achieves an ROC area of 0.93, a sensitivity of 0.67, a specificity of 0.94, a precision of 0.66, and a recall of 0.67. Without height and weight variables, the ROC area is 0.86, the sensitivity is 0.55, the specificity is 0.92, the precision is 0.55, and the recall is 0.55. The neural network classification model with all variables presents an ROC area of 0.99, a sensitivity of 0.94, a specificity of 0.99, a precision of 0.94, and a recall of 0.94. Without height and weight variables, the ROC area is 0.86, the sensitivity is 0.61, the specificity is 0.94, the precision is 0.60, and the recall is 0.61. The random forest model with height and weight variables included presents an ROC area of 1.00, a sensitivity of 0.96, a specificity of 0.99, a precision of 0.96, a recall of 0.96, an F-score of 0.96, a Matthews correlation coefficient (MCC) of 0.95, and a precision–recall curve (PRC) of 0.99. Without height and weight variables, the ROC area is 0.98, the sensitivity is 0.87, the specificity is 0.98, the precision is 0.88, the recall is 0.87, the F-score is 0.87, the MCC is 0.85, and the PRC is 0.92 (Table 2).

**Table 2:** Comparison of performance measurements of various machine learning models with and without height and weight variables.

Model, N=2,111		ROC area	Sensitivity	Specificity	Precision	Recall	F-Score	MCC	PRC
OneR (age)									
	With height and weight	0.81	0.67	0.94	0.67	0.67	0.67	0.61	0.51
	Without height and weight	0.67	0.44	0.90	0.42	0.44	0.40	0.33	0.28
Logistic regression									
	With height and weight	0.98	0.84	0.97	0.84	0.84	0.84	0.81	0.88
	Without height and weight	0.91	0.66	0.94	0.65	0.66	0.64	0.59	0.66
Decision tree									
	With height and weight	0.98	0.94	0.99	0.94	0.94	0.94	0.93	0.91
	Without height and weight	0.90	0.79	0.96	0.79	0.79	0.79	0.75	0.73
Random forest									
	With height and weight	1.00	0.96	0.99	0.96	0.96	0.96	0.95	0.99
	Without height and weight	0.98	0.87	0.98	0.88	0.87	0.87	0.85	0.92
SVM									
	With height and weight	0.96	0.85	0.97	0.85	0.85	0.85	0.82	0.79
	Without height and weight	0.86	0.63	0.94	0.63	0.63	0.62	0.56	0.53
KNN									
	With height and weight	0.90	0.82	0.97	0.82	0.82	0.82	0.79	0.72
	Without height and weight	0.88	0.78	0.96	0.78	0.78	0.78	0.75	0.67
Naive Bayes									
	With height and weight	0.93	0.67	0.94	0.66	0.67	0.67	0.61	0.72
	Without height and weight	0.86	0.55	0.92	0.55	0.55	0.52	0.47	0.57
Neural network									
	With height and weight	0.99	0.94	0.99	0.94	0.94	0.94	0.93	0.97
	Without height and weight	0.86	0.61	0.94	0.60	0.61	0.60	0.54	0.58

ROC area: area under the receiver operating characteristic curve; MCC: Matthews correlation coefficient; PRC: precision–recall curve; OneR: one rule; SMV: support vector machine; KNN: K-nearest neighbors.

## DISCUSSION

The fact that 50.6% of 2,111 people are male and 49.4% are female and the number of people in the seven groups in the BMI classification, which is the outcome variable, is almost equal increases the confidence in the results of the study. By determining which ML model achieves the highest performance based on daily lifestyle data without knowing height and weight values, we are offering a novel approach to combating obesity.

Some gynecological diseases may increase the risk of obesity^21^. Medical and surgical interventions and lifestyle alterations are also effective in obesity management. Bariatric surgery improves health outcomes and reduces long-term healthcare costs, despite initial high costs^22,23^. Bariatric surgery may be considered for infertility in women with morbid obesity^24^. Liraglutide, in conjunction with intragastric balloon placement, results in better weight loss than intragastric balloon placement alone, and there is no considerable difference in the results between males and females^25^. ML studies on obesity, which have various treatment alternatives, have become more critical.

A review of childhood obesity discusses the potential of ML to improve traditional prevention and treatment methods. The analysis shows that ML has significant potential to enhance strategies for managing pediatric obesity^26^. The study used ML techniques to predict metabolic syndrome, focusing on SVM and decision tree models. The findings demonstrated that SVM achieved better performance with a sensitivity of 0.774 and a specificity of 0.74, compared to the decision tree, which recorded a sensitivity of 0.76 and a specificity of 0.72^27^. In our study, although the SVM was not the most successful model, it still achieved a sensitivity of 0.63 and a specificity of 0.94 for obesity classification without using height and weight variables.

The analysis with a similar dataset also shows volumes about the need to perform data preprocessing in an ML analysis, specifically data integration and cleaning. Out of the models tried out, which include SVM, random forests, and decision trees, the random forest model was the most accurate, with a 96% accuracy rate^28^. The above study used the same dataset as the current study but with different methods and results. BMI is one of the main targets for prediction, and it can be obtained based on height and weight; however, we did not include these variables in the dataset to make the objective for ML models more attractive. This exclusion resulted in the expected reduction of prediction accuracy. The OneR method identified weight as the most significant variable; nevertheless, age was the most critical factor when height and weight were excluded. As for the differences between the two studies, our work demonstrates methodological and result-oriented variation even when working with the same dataset.

## CONCLUSION

In the clinical approach to obesity, which is now treatable, ML algorithms can be incorporated alongside medical algorithms. Our model successfully predicted BMI with 87% sensitivity, 99% specificity, and 88% precision, even without height and weight information. Further randomized controlled clinical trials are needed for more definitive results.
